# Toceranib phosphate-associated nephrotic syndrome in a dog: a case report

**DOI:** 10.1186/s12917-021-02850-9

**Published:** 2021-04-07

**Authors:** Shannon M. Remerowski, Chamisa L. Herrera, Lindsay L. Donnelly

**Affiliations:** 1 Department of Veterinary Medicine and Surgery, College of Veterinary Medicine, University of Missouri, 900 East Campus Drive, Columbia, MO 65211 USA; 2 Boundary Bay Veterinary Specialty Hospital, 4176 Meridian Street, Bellingham, WA 98226 USA

**Keywords:** Toceranib phosphate, Dog, Nephrotic syndrome, Mast cell tumor, Proteinuria

## Abstract

**Background:**

Nephrotic syndrome (NS) is rare in dogs and is characterized by concurrent clinical findings of proteinuria, hyperlipidemia, hypoalbuminemia, and edema. NS has been reported in humans receiving tyrosine kinase inhibitors (TKI) and in dogs receiving masitinib. This is the first report of NS in a dog receiving toceranib phosphate.

**Case presentation:**

An 8-year-old, female, spayed Labrador retriever was diagnosed with a 10 cm mast cell tumor on the left lateral abdomen. After completion of a 12-week vinblastine and prednisone protocol, she began treatment with toceranib phosphate (2.6 mg/kg by mouth, every other day). Proteinuria was documented prior to starting toceranib. On day 426 after diagnosis (day 328 of toceranib phosphate treatment), the dog was evaluated for diarrhea, lethargy and anorexia. On physical examination, dependent edema was noted on the ventral chest and abdomen, and sterile neutrophilic inflammation was aspirated from a 2.3 cm splenic nodule. The following laboratory values were reported: albumin < 1.5 g/dL; cholesterol 378 mg/dl and urine protein to creatinine ratio of 3.79. The patient was diagnosed with NS, and treatment with toceranib phosphate was discontinued. Low-dose aspirin was started in addition to an increased dosage of enalapril (0.47 mg/kg q12hr). No other therapy was instituted. The dog improved clinically, and laboratory values returned to near normal over the 8-week follow-up. She was euthanized 1399 days after discontinuing toceranib phosphate with progressive disease.

**Conclusions:**

Nephrotic syndrome is a potential adverse event associated with the drug toceranib phosphate which may be reversible with discontinuation of treatment. Careful monitoring of urine protein, serum biochemistry, blood pressure and patient weight is advisable during treatment with toceranib phosphate.

## Background

Nephrotic syndrome (NS) is the simultaneous development of hypoalbuminemia, hypercholesterolemia, proteinuria and edema or ascites and is a sequela of glomerular disease. NS has been described in human cancer patients receiving tyrosine kinase inhibitors (TKIs) [1–5]. Specifically, sunitinib is a molecule closely related to toceranib that has been associated with hypertension, minimal change disease (MCD), collapsing Focal Segmental Glomerulosclerosis (cFSGS) and nephrotic syndrome [6–9]. Toceranib phosphate (Zoetis, Florham Park, New Jersey) is a veterinary TKI approved by the FDA for treatment of canine Patnaik grade II and III cutaneous or recurrent mast cell tumors and has reported activity against multiple malignancies. The primary mechanism of action is small molecule inhibition of vascular endothelial growth factor receptor 2 (VEGFR2), platelet-derived growth factor receptor- ß (PDGFR-ß), and Kit. Adverse renal effects of toceranib phosphate administration include azotemia, protein losing nephropathy (PLN) and hypertension [10–12]. The development of NS in dogs receiving masitinib, a receptor tyrosine kinase inhibitor approved in Europe for use in dogs, has been described previously [13, 14]. This is the first report of nephrotic syndrome developing in a dog receiving toceranib phosphate.

## Case report

An 8-year-old, female, spayed Labrador retriever was presented to the University of Missouri Veterinary Health Center for treatment of a cytologically diagnosed mast cell tumor. On physical exam, a firm mass measuring 10 cm at longest diameter was present over the left lateral abdomen with concurrent ventral edema. The patient was lethargic with mild subjective hepatosplenomegaly. The remainder of the physical examination was unremarkable.

A complete blood count (CBC) revealed a mature neutrophilia (15.04 × 10^3/^uL ref.: 2.27–10.14), lymphopenia (0.51 × 10^3^/uL ref.: 0.76–4.23) and eosinopenia (0.00 × 10^3^/uL ref.: 0.08 to 1.1). Abnormalities on a serum chemistry panel included: hypoalbuminemia (2.6 g/dL ref.: 2.9–4), hyperglobulinemia (4.2 g/dL ref.: 2.2–3.6), and hypocalcemia (9.0 mg/dL ref.: 9.2–11.3). A urine sample was not obtained. Tumor grading and staging were recommended and declined.

Systemic treatment with vinblastine was started, and the dog received 2 mg/m^2^ intravenously on days 0, 14, 21, 28, 42, 56, 72 and 84 for a total of 8 doses. A CBC was performed prior to each vinblastine administration. Except for a neutropenia on day 7 which delayed dose 2 until day 14, there were no significant complications. During this time, the following medications were administered: prednisone (1.88 mg/kg PO q24h from day 0 to 21; 1.5 mg/kg PO q24h from day 21 to 28; 1.0 mg/kg PO q24h from day 28 to day 98; 0.5 mg/kg PO q24h from day 98 to day 106), famotidine (0.9 mg/kg PO q24h), diphenhydramine (1.8 mg/kg PO q24h), tramadol (1.8 mg/kg PO q8-12h, as needed), and maropitant (2.2 mg/kg PO q24h, as needed). By day 42 (vinblastine dose 5), the mass on her left lateral abdomen was 2 cm in diameter, an 80% reduction in longest length, and remained stable for the remainder of vinblastine treatment.

After completion of the 12-week vinblastine protocol (day 98), restaging was performed: CBC, serum chemistry, urinalysis, urine protein-to-creatine ratio (UPC), abdominal ultrasound and cytology of the liver. The CBC was unremarkable, and the serum chemistry and urinalysis revealed the following abnormalities, in addition to those summarized in Table 1: hypochloremia (107 mEq/L ref.: 108–117), elevated anion gap (24 mEq/L ref.: 13–22), elevated ALT (201 U/L ref.: 9–58), elevated alkaline phosphatase (ALP; 914 U/L ref.: 5–129), and elevated gamma glutamyl transferase (GGT; 33 U/L ref.: 0–5). The USG was 1.010 with a UPC of 6.48. The voided urine sediment was inactive. 4DX snap revealed all tests below detectable limits. An abdominal ultrasound was unremarkable except for mottled hepatic parenchyma. Cytologic evaluation of liver aspirates was consistent with steroid hepatopathy, and no evidence of mast cell infiltration was identified. Surgery, radiation, and/or medical management with toceranib phosphate were discussed. Treatment with toceranib phosphate was elected. Treatment was initiated at 65 mg (2.6 mg/kg PO EOD) in addition to enalapril (0.4 mg/kg PO q24h) for management of the PLN. Recheck exam, CBC, serum chemistry, and urinalysis were recommended to occur at 4–6 week intervals either with the local veterinarian or the University of Missouri Veterinary Health Center. Visits to the University of Missouri were required to occur at least every 90 days. Available clinical assessments including albumin, cholesterol, UPC, BUN, and creatinine are summarized in Table 1. At the day 120 recheck (day 22 of toceranib phosphate treatment), the mass measured 1.5 cm and remained stable for the duration of treatment with toceranib phosphate.

**Table 1:** Relevant laboratory values during treatment demonstrating criteria for NS developing 328 days after starting toceranib phosphate

	Relevant laboratory values during treatment with toceranib phosphate								
**Days post-presentation** (Days post-toceranib phosphate start)	**98** (0)	**120** (22)	**182** (84)	**262** (164)	**331** (233)	**426** (328)	**434** (336)	**449** (351)	**477** (379)
Albumin (g/dL) ref.: 2.9–4.0	2.8	2.6	3.2	3.2	2.7	< 1.5	1.8	2.6	2.8
Cholesterol (mg/dL) ref.: 133–338	353	317	274	359	233	378	x	x	x
UPC ref.: < 0.5	6.48	2.03	x	x	x	3.79	2.15	1.7	0.84
Edema	–	–	–	–	–	+	–	–	–
BUN (mg/dL) ref.: 8–28	12	x	16	11	14	21	20	18	17
Creatinine (mg/dL) ref.: 0.6–1.6	0.6	x	0.9	1.0	1.1	1.1	0.9	0.9	0.9

x indicates parameter not measured, − indicates parameter not present

On day 399 (day 301 of toceranib phosphate treatment), the dog developed acute diarrhea, inappetence and lethargy. A toceranib phosphate drug holiday was recommended and metronidazole (15 mg/kg PO q12h) was prescribed for seven days. One week later, stool consistency had improved, but lethargy and inappetence persisted. On day 413 (day 315 of toceranib phosphate treatment), two weeks after stopping toceranib phosphate, the dog’s appetite and demeanor was improved. Treatment resumed at the previous dose of 65 mg. Six days later, she, again, became inappetent and lethargic.

On day 426 (day 328 of toceranib phosphate treatment), the dog presented for evaluation. On physical examination, her left flank mast cell tumor measured 1.5 cm. She had lost 3.5 kg from the last oncology recheck 90 days prior, resulting in an unintentionally increased toceranib phosphate dosage of 3.02 mg/kg. At that time, a mass was palpated on the spleen, and dependent ventral edema was noted. The dog was normothermic. A CBC, serum chemistry, urinalysis, UPC, doppler blood pressure, abdominal ultrasound and cytology of the splenic mass were performed. A moderate, regenerative anemia was present (Hematocrit: 28% ref.: 37–57%). Significant abnormalities on serum chemistry and urinalysis included marked hypoalbuminemia, hypercholesterolemia and proteinuria (Table 1). The dog was hypertensive with a systolic blood pressure of 178 mmHg. Cytology of the 2.3 cm splenic mass revealed sterile, suppurative inflammation with some degenerate change. Surgical removal of the spleen was not recommended due to the patient’s poor candidacy for anesthesia. Due to development of NS, toceranib phosphate was discontinued. Aspirin was prescribed (0.93 mg/kg PO q24 hr), and the dosage of enalapril was increased (0.47 mg/kg PO q12h). No other medications were prescribed.

The dog was seen for rechecks at 1 week, 3 weeks and 8 weeks post-diagnosis with NS. At each of these rechecks, a CBC, renal chemistry profile and UPC were conducted. The splenic mass was no longer palpable at the 1-week recheck; an ultrasound was not performed, and no further treatment of the splenic mass was pursued. Within 8 weeks of discontinuing toceranib phosphate, dependent edema resolved, UPC returned to near-normal and albumin levels significantly improved. (Table 1). Cholesterol was not rechecked. No further treatment was pursued for her mast cell tumor. She was euthanized 1825 days after diagnosis (1399 days after discontinuing toceranib phosphate) due to progressive disease of the original mast cell tumor in her left flank region.

## Discussion and conclusions

Although less common than gastrointestinal adverse events, renal adverse events occur in a significant subset of dogs receiving TKIs. Proteinuria is a relatively common finding in dogs with cancer in general [15, 16]. Data from a 2016 study on hypertension and proteinuria in toceranib phosphate-treated dogs found that dogs with cancer had an elevated UPC relative to dogs without cancer at baseline, but this difference was not significant [12]. PLN has been documented to occur in up to 24% of dogs receiving toceranib phosphate, and most cases are successfully managed with dose reductions, angiotensin converting enzyme (ACE) inhibitors, and drug holidays [11]. A study on the safety and efficacy of masitinib showed that dogs with pre-existing renal disease were more likely to develop severe renal adverse effects, including nephrotic syndrome, while taking the drug [13]. This has not been conclusively demonstrated with toceranib phosphate.

Most renal adverse events associated with administration of toceranib phosphate are mild. In this case, a PLN with a UPC of 6.48 was documented prior to starting toceranib phosphate, and risk of further renal injury was discussed. Ultimately, non-invasive treatment with toceranib phosphate was elected, and the tumor was well-controlled beyond the 328 days of toceranib administration. ACE inhibition was instituted, and subsequent UPC performed on day 22 of toceranib phosphate treatment was 2.03. Dipstick urine protein was measured on days 84, 164 and 233 of toceranib phosphate treatment which were all negative or + 1, suggesting that the patient’s proteinuria likely improved for the majority of toceranib phosphate treatment. However, given the lack of objective urine protein measurements, this cannot be confirmed.

In this case, it appears that NS was reversible with cessation of toceranib phosphate. Marked improvement in proteinuria and hypoalbuminemia with resolution of edema after cessation of toceranib phosphate treatment supports that NS was likely an adverse event related to TKI therapy. Other than discontinuing toceranib phosphate, the only treatment this patient received was increasing enalapril dosing from SID to BID. By the 8-week recheck, the patient’s UPC was 0.84, and her hypoalbuminemia had improved markedly. This is consistent with the reported response of the dog that developed NS while being treated with masitinib [14]. In humans, most cases of NS resolved with discontinuation of TKI therapy or change to an earlier generation TKI that did not target VEGF [2, 5, 17]. However, renal dysfunction was irreversible in some cases [4, 7]. In those cases, length of TKI therapy with concomitant persistent proteinuria and cumulative glomerular damage was hypothesized to play a role. In the case reported here, NS developed after nearly a year of treatment with toceranib phosphate, and she had proteinuria prior to starting the drug. Pre-existing PLN likely increases the risk of NS secondary to TKIs.

Rechecks at the referral institution and dosage adjustments were performed every 3 months. Between referral visits, recheck exams and laboratory assessments were performed with the local veterinarian. Ideally, rechecks and dosage adjustments for dogs receiving tyrosine kinase inhibitors are performed every 4–6 weeks. The dog lost 3.4 kg in the 3 months prior to NS diagnosis. Perhaps gastrointestinal signs caused the weight loss, or gastrointestinal signs and NS may have been a result of the relative dose increase of toceranib phosphate. The dosage of toceranib phosphate at the time of NS development was 3.02 mg/kg which was an increase of 16%. The labeled dosage of toceranib phosphate is 3.25 mg/kg, but a dosage range of 2.4 mg/kg to 2.9 mg/kg EOD was found to minimize adverse effects while still reaching a plasma concentration in excess of the minimum concentration required for target inhibition [18].

Interestingly, the dog was diagnosed with a 2.3 cm splenic mass during the same visit that confirmed NS. Cytologic evaluation revealed sterile suppurative inflammation. No organisms were identified on cytology, but degenerate neutrophils were observed suggesting infection may have been present. No antimicrobial therapy was instituted, and this mass never caused clinical signs. The potential impact of splenic inflammation on the clinical picture was considered, but given the lack of fever, inflammatory leukogram, abdominal fluid or decline in patient status without treatment, the contribution is presumed to be minimal. In humans, secondary bacterial infections are a common complication of NS, particularly in children [19–21]. In one study, 40% of children hospitalized with NS had at least 1 infection [20]. Although infection has been reported in adults with NS, less data is available on incidence and treatment [22]. In the veterinary literature, there are no reports of secondary bacterial infection in patients with NS [23, 24]. Based on human studies, it is possible that splenic inflammation or infection was a complication of NS in this patient.

The mechanism for PLN development in patients being treated with TKIs is not well understood but is hypothesized to involve podocyte damage. Renal biopsies from humans who developed glomerular disease during TKI therapy showed evidence of podocytopathies, including focal segmental glomerulosclerosis (FSGS) and minimal change disease (MCD). MCD is a leading cause of NS development in children [6, 25]. Inhibition of tyrosine phosphorylation of nephrin, a podocyte protein present in the slit diaphragm, may be implicated in the observed podocyte injury [6].

There are limited medical treatments available for management of biologically aggressive mast cell tumors, and toceranib phosphate is an easily-administered, non-invasive choice with an objective response rate of 43% [10]. Patients should be routinely screened for proteinuria, hypertension and renal insufficiency prior to beginning treatment with toceranib phosphate. Careful monitoring of blood pressure, UPC and serum chemistry is indicated throughout treatment. Furthermore, frequent monitoring of patient weight with dose adjustments, as necessary, to remain in the range of 2.4 mg/kg to 2.9 mg/kg EOD is advisable.

## Data Availability

All relevant data are within this paper. The data generated during the current case study are available from the corresponding author on reasonable request.

## Abbreviations

NS: Nephrotic syndrome
TKI: Tyrosine kinase inhibitor
PLN: Protein losing nephropathy
PO: Per os
EOD: Every other day
FDA: Food and Drug Administration
CBC: Complete blood count
FSGS: Focal segmental glomerulosclerosis
MCD: Minimal change disease
UPC: Urine protein-to-creatinine ratio
ACE: Angiotensin converting enzyme
ALT: Alanine transaminase
ALP: Alkaline phosphatase
GGT: Gamma-glutamyl transferase
BUN: blood urea nitrogen
